# Synthesis and Photophysical Properties of 2-Aryl-6,8-bis(arylethenyl)-4-methoxyquinolines 

**DOI:** 10.3390/molecules171214186

**Published:** 2012-11-30

**Authors:** Tebogo Ankie Khoza, Marole Maria Maluleka, Neliswa Mama, Malose Jack Mphahlele

**Affiliations:** 1 Department of Chemistry, College of Science, Engineering and Technology, University of South Africa, P.O. Box 392, Pretoria 0003, South Africa; 2 Department of Chemistry, Nelson Mandela Metropolitan University, P.O. Box 77000, Port Elizabeth 6031, South Africa

**Keywords:** 1-(2-amino-3,5-dibromophenyl)-3-aryl-2-propen-1-ones, 2-aryl-6,8-dibromo-2,3-dihydroquinolin-4(1*H*)-ones, 2-aryl-6,8-dibromoquinolin-4(1*H*)-ones, 2-aryl-6,8-dibromo-4-methoxyquinolines, Suzuki-Miyaura cross-coupling, 2-aryl-6,8-bis(2-aryl-ethenyl)-4-methoxyquinolines, photophysical properties

## Abstract

Iodine-methanol mediated oxidative-aromatization of 2-aryl-6,8-dibromo-2,3-dihydroquinolin-4(1*H*)-ones afforded the corresponding 2-aryl-6,8-dibromo-4-methoxy-quinolines in high yield and purity. The isomeric 1-(2-amino-3,5-dibromophenyl)-3-aryl-2-propen-1-ones reacted with iodine in methanol afford in a single pot operation the corresponding 2-aryl-6,8-dibromo-4-methoxyquinoline (major) and 2-aryl-6,8-dibromoquinolin-4(1*H*)-one (minor) products that were separated in sequence by column chromatography on silica gel. Suzuki-Miyaura cross-coupling of the 6,8-dibromo-4-methoxyquinoline derivatives with excess arylvinylboronic acids afforded the corresponding 2-aryl-6,8-bis(2-arylethenyl)-4-methoxyquinolines. The absorption and fluorescence properties of these compounds were also determined.

## 1. Introduction

2-Aryl-2,3-dihydroquinolin-4(1*H*)-ones are valuable precursors for the synthesis of 2-arylquinolin-4(1*H*)-ones and 4-substituted quinoline derivatives. The heterocyclic ring of 2-aryl-2,3-dihydro- quinolin-4(1*H*)-ones enables different degrees of unsaturation via C_2,3_ dehydrogenation to afford the potentially tautomeric NH-4-oxo derivatives [1,2,3] or oxidative aromatization to afford the 4-alkoxy-2-arylquinolines [3,4,5,6,7,8]. The 2-arylquinolin-4(1*H*)-ones formed through dehydrogenation using either iodobenzene diacetate under basic conditions in methanol [1] or thallium(III) *p*-tolylsulfonate in dimethoxyethane [3] were found to undergo base-mediated alkylation to afford the corresponding 4-alkoxy-2-arylquinolines. The tautomeric equilibrium between the incipient quinolin-4(1*H*)-one and its quinolinol isomer often leads to mixtures of *N*-alkylquinolinones and *O*-alkylquinolines, particularly when primary alkyl halides such as iodomethane are used as alkylating reagent [9]. An indirect, but sure-fire approach which involves oxidative aromatization of the 2-arylquinolin-4-(1*H*)-ones with phosphoryl halides to afford the corresponding 2-aryl-4-chloro/bromoquinolines, followed by dehalogenoalkoxylation, affords the 4-alkoxyquinolines, albeit in moderate yields [10]. The most convenient direct syntheses of 4-methoxy-2-arylquinoline derivatives developed to-date involves oxidative aromatization of 2-aryl-2,3-dihydroquinolin-4(1*H*)-ones using oxidative reagents such as thallium(III) nitrate [4] or [hydroxyl(tosyloxy)iodo]benzene [5] in trimethyl orthoformate in the presence of perchloric acid as catalyst, molecular iodine in refluxing methanol [3,6] or FeCl_3_·6H_2_O in methanol [7]. We required polysubstituted quinolines in which an electron-acceptor 2-aryl-4-methoxy-quinoline framework is linked to the π-conjugated spacer through positions 6 and 8 to comprise a donor-π-acceptor system. Such derivatives would not be easily accessible via the known classical methods such as the Skraup, Friedlander and Doebner-von Miller reactions. The 2-aryl-6,8-dibromo-4-methoxyquinolines appeared suitable candidates for metal catalyzed cross-coupling to afford the requisite donor-π-acceptor systems. This prompted us to prepare the 2-aryl-6,8-dibromo-4-methoxyquinolines to serve as substrates for the Suzuki-Miyaura cross-coupling with arylvinylboronic acids to afford the requisite polysubstituted quinolines with potential photophysical properties.

## 2. Results and Discussion

### 2.1. Synthesis of the 2-Aryl-6,8-dibromo-2,3-dihydrquinolin-4(1H)-ones

**Scheme 1:**
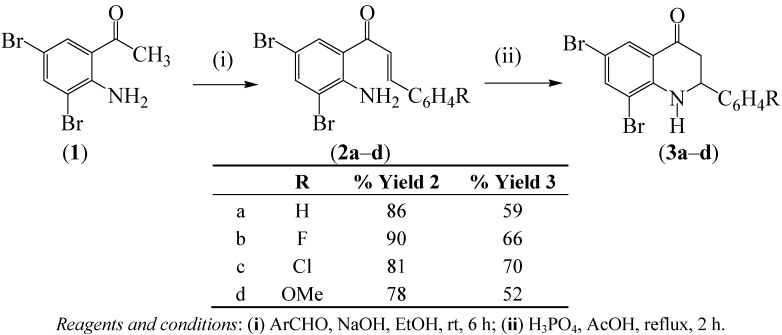
Synthesis of 2-aminochalcones **2a**–**d** and their transformation into **3a**–**d**.

The known 2-aryl-6,8-dibromo-2,3-dihydroquinolin-4(1*H*)-ones [3,11] appeared to be suitable substrates for oxidative-aromatization to afford the requisite 2-aryl-6,8-dibromo-4-methoxyquinolines. The 2-aryl-2,3-dihydroquinolin-4(1*H*)-ones are conveniently accessible via acid- or base-mediated cyclization of the corresponding substituted 2-aminochalcones [12,13,14]. Base-promoted Claisen-Schmidt aldol condensation of 1-(2-amino-3,5-dibromophenyl)ethanone with benzaldehyde derivatives at low temperature (0–5 °C) previously afforded the corresponding 1-(2-amino-3,5-dibromophenyl)-3-aryl-2-propen-1-ones after 35–40 h [11]. In this investigation, we condensed 1-(2-amino-3,5-dibromophenyl)ethanone (**1**) with substituted benzaldehyde derivatives and found the reaction to proceed smoothly and rapidly at room temperature to afford the corresponding 1-(2-amino-3,5-dibromophenyl)-3-aryl-2-propen-1-ones **2a**–**d** in high yield and purity within 6 h (Scheme 1). Compounds **2a**–**d** were, in turn, subjected to acid-mediated cyclization with orthophosphoric-acetic acid mixture to afford the isomeric 2-aryl-6,8-dibromo-2,3-dihydroquinolin-4(1*H*)-ones **3a**-**d** (Scheme 1). The 2-aryl-6,8-dibromo-2,3-dihydroquinolin-4(1*H*)-ones **3a**–**d** were recently found to exhibit *in vitro* activity against the MCF-7 breast cancer cell line [11]. The 2-aryl-2,3-dihydroquinolin-4(1*H*)-ones were previously found to serve as antitumour agents by virtue of their capacity to bind to tubulin and thereby function as spindle toxins [15,16]. Moreover, some derivatives have also been found to act as microRNA inhibitors and also to control cell proliferation by altering the microRNA levels [17].

We also obtained crystals of **3b** of suitable quality for X-ray diffraction and the geometry of these novel systems was thus further confirmed by X-ray diffraction (Figure 1) [18]. The six-membered ring is in a half-chair arrangement, with the 2-aryl ring deformed out of the plane of the quinolinone ring.

**Figure 1:**
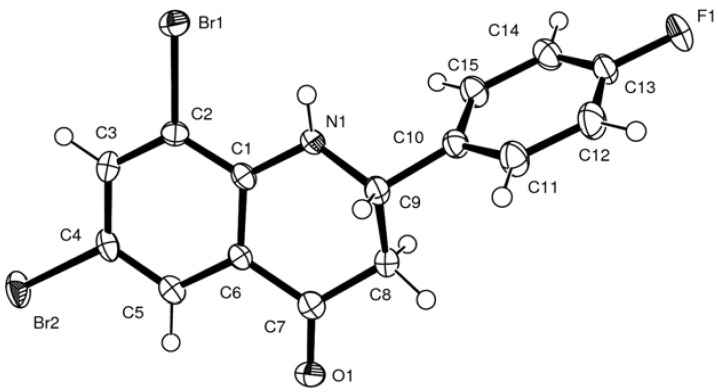
X-ray crystal structure of 6,8-dibromo-2-(4-fluorophenyl)-2,3-dihydroquinolin-4(1*H*)-one (**3b**) showing crystallographic numbering. For clarity, hydrogen atoms are not labeled.

### 2.2. Synthesis of the 4-Alkoxy-2-arylquinolines

#### 2.2.1. Oxidative-aromatization of the 2,3-Dihydroquinolin-4(1*H*)-ones

**Scheme 2:**
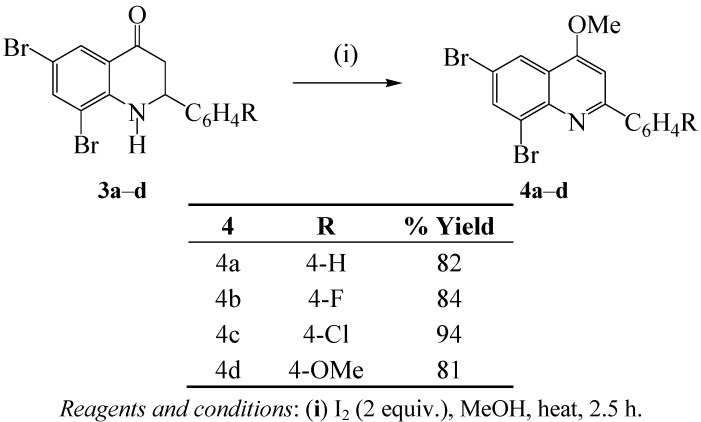
Iodine/methanol-mediated oxidative aromatization of **3a**–**d**.

With the intent to synthesize 2,6,8-triaryl-4-methoxyquinolines for further transformation, we subjected systems **3** to oxidative aromatization using molecular iodine (2 equiv.) in methanol under reflux. We isolated the corresponding 2-aryl-6,8-tribromo-4-methoxyquinolines **4a**–**d** in high yield and purity without the need for column chromatographic separation (Scheme 2). The analogous naturally occurring 4-methoxy-2-phenylquinoline and its 2-(methylene-dioxy-phenyl) derivative have been found to exhibit inhibitory activity against *Mycobacterium tuberculosis* H_37_Rv [19].

#### 2.2.2. Oxidative-cyclization of 1-(2-Amino-3,5-dibromophenyl)-3-aryl-2-propen-1-ones

**Scheme 3:**
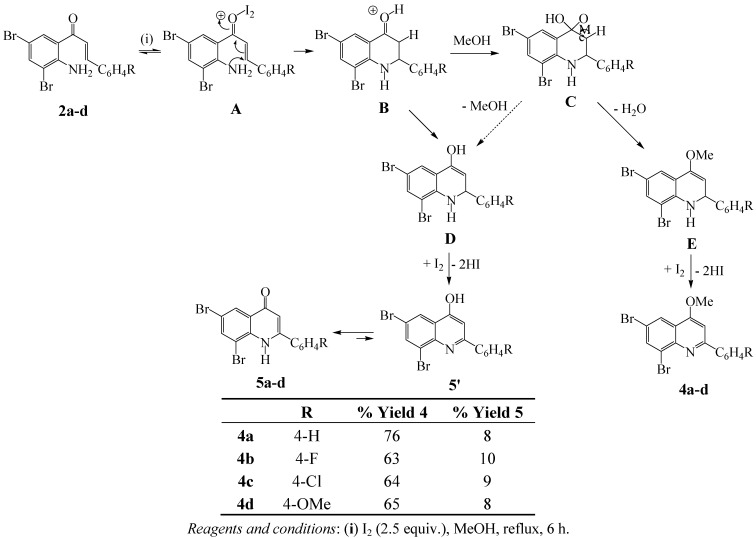
Direct one-pot iodine-methanol-mediated oxidative-cyclization of **2**.

A direct one-pot synthesis of the 2-aryl-4-methoxyquinolines from the isomeric 2'-aminochalcones involving FeCl_3_.6H_2_O mediated oxidative cyclization in methanol has been described in the literature [20]. However, according to the authors oxidizing agents such as Mn(OAc)_2_, CAN, PCC, NBS, Co(NO_3_)_2_–K_2_S_2_O_8_ and iodine in methanol all failed to effect oxidative cyclization of the 2'-aminochalcones [20]. We envisioned that the electrophilic and oxidative potential of iodine would promote cyclization and oxidative aromatization of systems **2a**–**d** to afford compounds **4a**–**d**. Based on this hypothesis, we subjected the 2'-aminochalcones **2** to iodine (2.5 equiv.) in methanol under reflux. Interestingly in our case, we isolated in each case by column chromatography on silica gel the corresponding (major) 2-aryl-6,8-dibromo-4-methoxyquinolines **4a**–**d** and (minor) 2-aryl-6,8-dibromo-quinolin-3(1*H*)-one derivatives **5a**–**d** in sequence (Scheme 3). We rationalize the formation of products **4a**–**d** and **5a**–**d** as a consequence of the initial iodine-mediated cyclization to form the incipient 2-aryl-2,3-dihydroquinolin-4(1*H*)-one. Protonation of the 2-aryl-2,3-dihydro-quinolin-4(1*H*)-one occurs to afford **B**, which may undergo enolization to **D** or nucleophilic addition by methanol to yield a hemiacetal **C**. Extrusion of water from **C** yields the enolether intermediate **E**, which then undergoes iodine-promoted oxidative aromatization to afford **4**. Iodine-promoted oxidative aromatization of the enol derivative **D** (which may also be a consequence of extrusion of methanol from **C**), on the other hand, affords the potentially tautomeric quinolinol derivatives **5**'. The 4-quinolinol *versus* 4-quinolinone tautomeric equilibrium has been found by spectroscopic and X-ray crystallography means to favour the preponderance of the NH-4-oxo tautomer **5** in solution, gas and solid state [21]. Whereas systems **5b**–**d** are described in this investigation for the first time, the 6,8-dibromo-2-phenylquinolin-4(1*H*)-one **5a** was previously isolated as a minor product from the reaction of **3a** with excess bromine (4 equiv.) in chloroform [22].

### 2.3. Suzuki-Miyaura Cross-Coupling of the 2-Aryl-6,8-dibromo-4-methoxyquinolines

Bromoquinolines are of interest to chemists as precursors for heterocyclic compounds with multifunctionality giving a wide variety of compounds through, e.g., couplings [23,24] and metal exchange reactions [25,26]. A one-pot borylation of 8-bromo/chloroquinolines with bis(pinacolato)-diboron and subsequent Suzuki-Miyaura cross-coupling of the resultant 8-quinoline-boronic acids with aryl halides, for example, previously afforded the 8-arylquinolines in high yields [23]. However, lack of selectivity was observed for 8-bromo-6-chloroquinoline with 1 equiv. of bis(pinacolato)diboron and the authors, in turn, used an excess of bis(pinacolato)diboron (2.2 equiv.) and phenylbromide to afford 6,8-diphenylquinoline in 94% yield [23]. Dibromoquinolines such as 5,7-dibromoquinoline and 8-benzyloxy-5,7-dibromoquinoline, on the other hand, undergo Suzuki cross-coupling with less or no selectivity compared to many of the other dibromoheteroaromatics [24,27]. Our initial trial to effect the Suzuki-Miyaura cross-coupling of 6,8-dibromo-4-methoxy-2-phenylquinoline (**1a**) and 4-fluoro-phenylvinylboronic acid (1 equiv.) using Pd(PPh_3_)_4_ as Pd(0) source and K_2_CO_3_ as a base in DMF-water (3:1, v/v) resulted in the recovery of the starting material. The failure of Pd(PPh_3_)_4_ to promote the cross-coupling is probably due to the inhibiting role of the extra PPh_3_ generated in the second equilibrium {*S*Pd(0)(PPh_3_)_3_ ⇄ *S*Pd(0)(PPh_3_)_2_ + PPh_3_ (*K*_2_/[PPh_3_] << 1); *S* = solvent} to afford the reactive low ligated 14-electron species (Pd(0)(PPh_3_)_2_) [28]. Conversely, the oxidative addition performed by the palladium(0) complex (Pd(0)(PPh_3_)_2_Cl^−^) generated by the reduction of dichlorobis-(triphenylphosphine)palladium(II) (PdCl_2_(PPh_3_)_2_) is reported to be more than 30 times faster than that performed from Pd(0)(PPh_3_)_4_ [28]. 

**Scheme 4:**
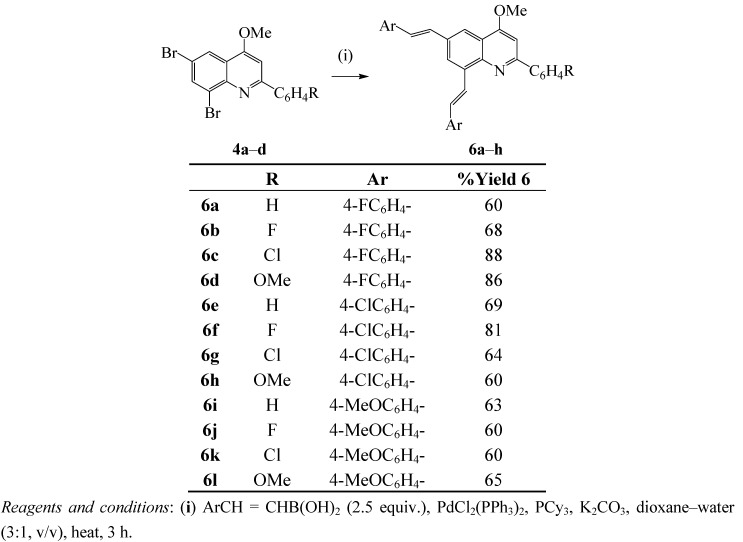
One-pot Suzuki-Miyaura cross-coupling of **4** with arylvinylboronic acids.

Application of dichlorobis-(triphenylphosphine)palladium(II) (PdCl_2_(PPh_3_)_2_ as Pd(0) catalyst source, on the other hand, led to incomplete conversion of **4a** to afford the cross-coupled product **6a** in 55% yield after 18 h. The prolonged reaction times and reduced yield prompted us to use PdCl_2_(PPh_3_)_2_–tricyclohexylphosphine catalyst complex in DMF-water (3:1, v/v) and an excess of arylboronic acid (2.5 equiv.) in the presence of K_2_CO_3_ as a base in analogy with the literature precedents [23,24,27] and we achieved complete conversion of **4a** to **6a** within 3 h. Alkylphosphine ligands, are known to coordinate with palladium and increase its electron density more so than arylphosphines and, in turn, accelerate the oxidative addition and reductive elimination steps in the catalytic cycle [28,29]. Extension of these reaction conditions to other dibromoquinolines **4** using 4-substituted phenyvinylboronic acids as coupling partners afforded the corresponding 2-aryl-6,8-bis(2-arylethenyl)-4-methoxyquinolines **6a**–**l** in high yield without the need for column chromatography (Scheme 4). 

The analogous aryl, alkenyl and alkynyl substituted quinoline derivatives have been found to serve as potent inhibitors of tyrosine kinase PDGF-RTK [30], and anti-retroviral agents [31] or LTD_4_ receptor antagonists [32]. The 4-alkoxy-3,6-diarylquinolines, on the other hand, are reported to exhibit potent and selective agonism of the somatostatin receptor subtype 2 (sst_2_) and to represent promising agents for the treatment of diabetic retinopathy and proliferative diseases [33]. Likewise, the 6- or 8-aryl substituted 2,4-dimethoxyquinolines were found to exhibit high activity against the agriculturally important nematode, *Haemonchus contortus* with potency comparable to that of the commercially available levamisole [34]. Systems **6a**–**l** are also analogues of the 2-aryl-3,4-bis(phenylethenyl) quinolines and the 3,4-bis(alkynyl)-2-arylquinolines with potential to serve as molecular organic materials in nanomaterials or building blocks for polyquinolines or quinoline-based copolymers with enhanced photonic and electronic properties [35].

### 2.4. Photophysical Property Studies of Systems *6*

Polysubstituted quinolines **6a**–**l** comprise an electron-deficient quinoline framework as an electron-acceptor linked to the 4-substituted aryl ring via a π-conjugated spacer to comprise a donor-π-acceptor system. Preliminary absorption and fluorescence properties of systems **6a**–**l** were determined in chloroform at room temperature.

#### 2.4.1. UV-Vis Absorption Properties of 4-Methoxyquinoline Derivatives **6**

The electronic absorption spectra of the 4-methoxyquinoline derivatives **6a**–**l** were acquired in CHCl_3_ and the compounds absorb in the region μ 310–390 nm (Figure 1). The absorption spectra of these compounds are characterized by intense absorption peaks near 310–350 nm and the less intense ones around 360–380 nm. The band in the region 310–350 nm is due to π–π* transition attributed to the conjugated quinoline ring of the molecule in analogy with the assignment for π-styrylquinolines [36], whereas the lowest energy band which is largely of charge transfer character is due to the 2-aryl group.

Both the absorption maxima and wavelength are influenced by the variation of substituents on the arylvinyl and the 2-aryl groups. Compounds **6d**, **6f**, **6i** and **6k** showed strong UV-*vis* absorption intensities at around 340 nm reflecting the following trend in intensity: **6d** > **6k** > **6i** > **6f**. A combination of the strong electron donating 2-(4-methoxyphenyl) group and moderately donating 4-fluorophenylvinyl substituents in **6d** seem to increase the electron density of the quinoline ring thus the π-π* transition. A combination of the relatively less donating 2-(4-chlorophenyl) group or 2-phenyl group and the two 4-methoxyvinyl substituents on the fused benzo ring also enhance the absorptivities for **6f** and **6i**, respectively. Enhanced intensity comparable to that of **6f** but at different wavelength is also observed for **6h** bearing the 2-(4-methoxyphenyl) group and the two 4-chlorophenylvinyl groups. Except for the 2-(4-methoxyphenyl) substituted derivative **6d**, it seems the presence of 4-fluorophenyl moieties in **6a**, **b** and **c** led to reduced absorption intensities than for derivatives bearing chloro atoms on the styryl groups. It appears the presence of the chloro atoms increase the electron affinity of the quinoline ring than the fluoro atoms. 

**Figure 1:**
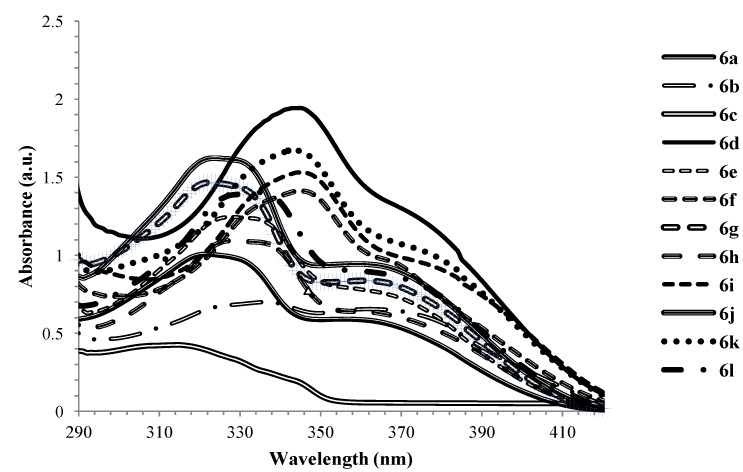
UV-Vis spectra of **6a**–**l** in CHCl_3_ (2.0 × 10^−5^ mol/L).

#### 2.4.2. The Fluorescence Properties of 4-Methoxyquinoline Derivatives

**Figure 2:**
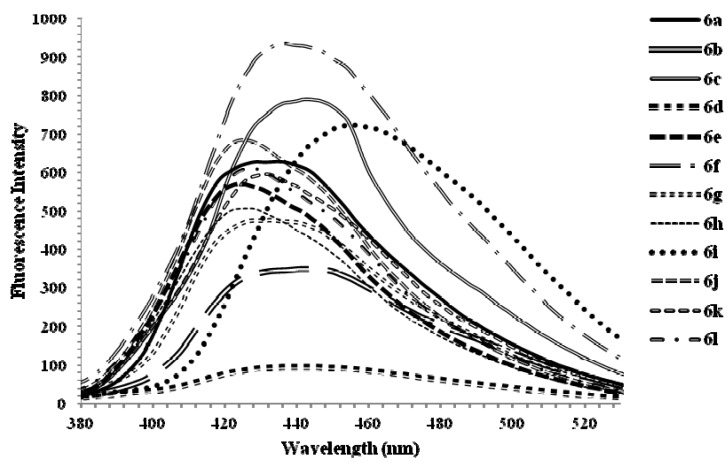
The fluorescence emission spectra of **6a**–**l** (excitation wavelength λ_ex_ = 355 nm) in CHCl_3_ (2.0 × 10^−7^ mol/L) at rt.

The fluorescence properties of compounds **6a**–**l** have been studied at room temperature in the moderately polar chloroform at the excitation wavelength of 355 nm (Figure 2). 

The fluorescence spectra of these compounds show similar pattern and are characterized by a single emission band in the region 410–490 nm, which are attributed to π-π* transition of the conjugated system. The fluorescence patterns of compounds **6a**–**l** are affected by the presence of the halo substituents on the styryl and 2-phenyl groups [37]. Systems **6c** and **6f** with mixed halides (F and Cl) on either the 2-phenyl or styryl groups show increased emission and slight bathochromic shifts. A combination of the 2-(4-methoxyphenyl) and 4-fluorophenylvinyl derivative **6d** significantly reduces the emission intensity than the other derivatives bearing the 4-fluorophenylvinyl substituents. Increased emission and pronounced red shift effect are also observed for **6i** bearing the 2-phenyl and 4-methoxystyryl groups. The increased intensities and bathochromic shifts are presumably the result of increased π-electron delocalization along the vinylene bridge and/or the 2-aryl group towards the electron-deficient quinoline ring. 

## 3. Experimental

### 3.1. General

Melting points were recorded on a Thermocouple digital melting point apparatus and are uncorrected. IR spectra were recorded as powders using a Bruker VERTEX 70 FT-IR Spectrometer with a diamond ATR (attenuated total reflectance) accessory by using the thin-film method. For column chromatography, Merck kieselgel 60 (0.063–0.200 mm) was used as stationary phase. NMR spectra were obtained as CDCl_3_ solutions using Varian Mercury 300 MHz NMR spectrometer and the chemical shifts are quoted relative to the solvent peaks. Low- and high-resolution mass spectra were recorded at an ionization potential of 70eV using Micromass Autospec-TOF (double focusing high resolution) instrument. The synthesis and characterization of substrate **1** have been described elsewhere [38]. The UV-vis spectra were recorded on a Perkin Elmer Lambda 35 UV/vis spectrometer while emission spectra were taken using a Perkin Elmer LS 45 fluorescence spectrometer.

### 3.2. Typical Procedure for the Synthesis of the 2-Amino-4,6-dibromochalcones *2a–d*

*1-(2-Amino-3,5-dibromophenyl)-3-phenyl-2-propen-1-one *(**2a**). A stirred mixture of 2-amino-3,5-dibromoacetophenone (**1**, 2.00 g, 6.80 mmol) and benzaldehyde (0.721 g, 6.80 mmol) and sodium hydroxide (0.60 g) in ethanol (30 mL) was stirred at room temperature for 6 h and then quenched with an ice-cold water. The resulting precipitate was filtered and then taken-up into chloroform. The chloroform solution was dried over MgSO_4_, filtered and then evaporated under reduced pressure to afford **2a** as a yellow solid (2.23 g, 86% yield), m.p. 120–123 °C (ethanol); ν_max_ (ATR) 688.9, 771.1, 974.0, 1186.1, 1336.5, 1514.9, 1560.6, 1592.5, 1644.8 3294.5, 3400.0, 3462.4 cm^−1^; δ_H_ (CDCl_3_) 6.92 (br s, 2H), 7.41–7.43 (3H), 7.48 (d, *J *15.6 Hz, 1H), 7.62–7.65 (m, 2H), 7.70 (d, *J* 1.8 Hz, 1H), 7.92 (d, *J* 15.6 Hz, 1H), 7.92 (d, *J* 1.8 Hz, 1H); δ_C_ (CDCl_3_) 106.1, 111.8, 120.7, 121.9, 128.5, 129.0, 130.6, 132.6, 134.7, 139.1, 144.7, 146.7, 146.8, 190.1; *m/z* 380 (100, MH^+^); HRMS (ES): MH^+^, found 379.9284. C_15_H_12_NO^79^Br_2_^+^ requires 379.9286.

*1-(2-Amino-3,5-dibromophenyl)-3-(4-fluorophenyl)-2-propen-1-one *(**2b**). A mixture of **1** (2.00 g, 6.80 mmol), 4-fluorobenzaldehyde (0.85 g, 6.80 mmol) and NaOH (0.60 g) in ethanol (30 mL) afforded **2b** as a yellow solid (2.45 g, 90%), m.p. 149–150 °C (ethanol) (Lit. 118–120 °C [11]).

*1-(2-Amino-3,5-dibromophenyl)-3-(4-chlorophenyl)-2-propen-1-one *(**2c**). A mixture of **1** (2.00 g, 6.80 mmol), 4-chlorobenzaldehyde (0.96 g, 6.80 mmol) and NaOH (0.60 g) in ethanol (30 mL) afforded **2c** as an orange solid (2.30 g, 81%), m.p. 145–148 °C (ethanol) (Lit. 124–128 °C [11]).

*1-(2-Amino-3,5-dibromophenyl)-3-(4-methoxyphenyl)-2-propen-1-one *(**2d**). A mixture of **1** (2.00 g, 6.80 mmol), 4-methoxybenzaldehyde (0.93 g, 6.80 mmol) and NaOH (0.60 g) in ethanol (30 mL) afforded **2d** as a yellow solid (2.25 g, 78%), m.p. 139–141 °C (ethanol) (Lit. 130–135 °C [11]).

### 3.3. Typical Procedure for the Synthesis of *3a–d*

A stirred mixture of **2a** (3.00 g, 7.87 mmol) and orthophosphoric acid (20 mL) in glacial acetic acid (40 mL) was heated under reflux for 2 h. The mixture was allowed to cool to room temperature and then quenched with an ice-cold water. The product was extracted into chloroform and the combined organic phases were washed thoroughly with saturated solution of sodium carbonate and then dried over MgSO_4_. The solvent was evaporated under reduced pressure and the residue was recrystallized from ethanol to afford **3a** as a light yellow solid (1.78 g, 59%), m.p. 135–136 °C (Lit. 131–133 °C [34]). The following products were prepared in this fashion: **3b** (1.97 g, 66%), m.p. 127–129 °C (Lit. 126–129 °C [35]); **3c** (2.10 g, 70%), m.p. 133–135 (Lit. 138–140 °C [11]) and **3d** (1.57 g, 52% yield), m.p. 143–145 °C (Lit. 149–151 °C [35]).

### 3.4. Typical Procedure for the Aromatization of *3a–d* with Iodine in Methanol

*6,8-Dibromo-4-methoxy-2-phenylquinoline* (**4a**). A stirred mixture of **3a** (1.00 g, 2.62 mmol) and iodine (1.33 g, 5.25 mmol) in methanol (20 mL) was refluxed for 3 hours. The mixture was quenched with an ice-cold solution of saturated sodium thiosulphate and the product was extracted into chloroform. The combined chloroform phases were dried over MgSO_4_, filtered and then evaporated under reduced pressure to afford **4a** as a white solid (0.84 g, 82%) m.p. 174–176 °C (ethanol); ν_max_ (ATR) 776, 868, 1002, 1219, 1357, 1477, 1576 cm^−1^; δ_H_ (CDCl_3_) 4.12 (s, 3H), 7.24 (s, 1H), 7.46–7.55 (m, 3H), 8.10 (d, *J *= 1.8 Hz, 1H), 8.20 (d, *J *= 1.5 Hz, 1H), 8.23 (d, *J *= 1.5 Hz, 1H), 8.28 (d, *J *= 1.8 Hz, 1H); δ_C_ (CDCl_3_) 56.1, 98.5, 118.3, 122.5, 124.1, 127.6, 128.8, 129.9, 136.2, 139.2, 144.8, 159.0, 162.1; *m/z* 394 (100%, C_16_H_12_NO^79^Br^81^Br^+^); HRMS (ES): MH^+^, found 391.9277. C_16_H_12_NO^79^Br_2_^+^ requires 391.9286.

*2,6,8-Dibromo-2-(4-fluorophenyl)-4-methoxyquinoline *(**4b**). A stirred mixture of **3b** (1.00 g, 2.50 mmol) and iodine (1.27 g, 5.01 mmol) in methanol (20 mL) afforded **4b **as a white solid (0.86 g, 84%) m.p. 172–175 °C (ethanol); ν_max_ (ATR) 718, 828, 1002, 1218, 1361, 1481, 1583 cm^−1^; δ_H_ (300 MHz, CDCl_3_) 4.08 (s, 3H), 7.12 (s, 1H), 7.18 (t, *J *= 8.4 Hz, 2H), 8.07 (d, *J *= 2.1 Hz, 1H), 8.16–8.23 (m, 3H); δ_C_ (75 MHz, CDCl_3_) 56.0, 98.0, 115.8 (d, ^2^*J*_CF_ = 21.6 Hz), 118.3, 122.1, 124.1, 129.4 (d, ^3^*J*_CF_ = 8.3 Hz), 135.2 (d, ^4^*J*_CF_ = 3.2 Hz), 136.3, 144.6, 157.7, 162.1, 164.1 (d, ^1^*J*_CF_ = 248.5 Hz); *m/z* 412 (100%, C_16_H_11_NO^79^Br^81^BrF^+^); HRMS (ES): MH^+^, found 409.9175. C_16_H_11_NO^79^Br_2_F^+^ requires 409.9151.

*6,8-Dibromo-2-(4-chlorophenyl)-4-methoxyquinoline *(**4c**). A stirred mixture of **3c** (0.89 g, 2.14 mmol) and iodine (1.09 g, 4.28 mmol) in methanol (20 mL) afforded **4c **as a white solid (0.85 g, 94%) m.p. 198–200 °C (ethanol); ν_max_ (ATR) 692, 820, 1000, 1093, 1219, 1357, 1437, 1479, 1588 cm^−1^; δ_H_ (CDCl_3_) 4.10 (s, 3H), 7.16 (s, 1H), 7.62 (d, *J* 8.7 Hz, 2H), 8.09 (d, *J* 2.1 Hz, 1H), 8.15 (d, *J *= 8.7 Hz, 2H), 8.26 (d, *J *= 2.1 Hz, 1H); δ_C_ (CDCl_3_) 56.1, 98.1, 118.6, 122.2, 124.1, 125.9, 128.8, 129.0, 136.1, 136.4, 137.5, 144.7, 157.6, 162.2; *m/z* 428 (100%, C_16_H_11_NO^37^Cl^79^Br_2_^+^ or C_16_H_11_NO^35^Cl ^79^Br^81^Br^+^); HRMS (ES): MH^+^, found 425.8974. C_16_H_11_NO^35^Cl^79^Br_2_^+^ requires 425.8974.

*6,8-Dibromo-4-methoxy-2-(4-methoxyphenyl)quinoline* (**4d**). A stirred mixture of **3d** (1.00 g, 2.42 mmol) and iodine (1.23 g, 4.84 mmol) in methanol (20 mL) afforded **4d** as a white solid (0.82 g, 81%) m.p. 193–194 °C (ethanol); ν_max_ (ATR) 1001, 1031, 1176, 1220, 1244, 1363, 1486, 1576 cm^−1^; δ_H_ (CDCl_3_) 3.88 (s, 3H), 4.06 (s, 3H), 7.02 (d, *J *= 9.0 Hz, 2H), 7.16 (s, 1H), 8.07 (d, *J *= 2.1 Hz, 1H), 8.18 (d, *J *= 9.0 Hz, 2H), 8.23 (d, *J *= 2.1 Hz, 1H); δ_C_ (CDCl_3_) 55.4, 56.0, 97.8, 114.2, 117.8, 122.0, 124.0, 125.7, 129.0, 131.7, 136.1, 144.8, 158.5, 161.3, 162.0; *m/z* 424 (100%, C_17_H_14_NO_2_^79^Br^81^Br); HRMS (ES): MH^+^, found 421.9378. C_17_H_14_NO_2_^79^Br_2_^+^ requires 421.9391.

### 3.5. Typical Procedure for the One-pot Cyclization and Aromatization of *2* with Iodine in Methanol

Preparation of 6,8-dibromo-4-methoxy-2-phenylquinoline (**4a**) and 6,8-dibromo-2-phenylquinolin-4(1H)-one (**5a**). A stirred mixture of **3a** (0.50 g, 1.32 mmol) and iodine (0.83 g, 3.28 mmol) in methanol (20 mL) was refluxed for 5 h. The mixture was quenched with an ice-cold solution of saturated sodium thiosulphate and the product was extracted into chloroform. The combined chloroform phases were dried over MgSO_4_, filtered and then evaporated under reduced pressure. The residue was purified by column chromatography on silica gel (40% ethyl acetate-hexane) to afford **4a** and **5a** in sequence.

*6,8-Dibromo-4-methoxy-2-phenylquinoline* (**4a**). White solid (0.390 g, 76%), *R*_f_ 0.77.

*6,8-Dibromo-2-phenylquinolin-4(1H)-one* (**5a**). White solid (0.04 g, 8%) m.p. 212–213 °C (Lit. 213 °C [22]), *R*_f_ 0.37; ν_max_ (ATR) 734, 843, 866, 1349, 1383, 1491, 1537, 1620, 3389 cm^−1^; δ_H_ (CDCl_3_) 6.60 (s, 1H), 7.56–7.59 (m, 3H), 7.66–7.71 (m, 2H), 7.96 (d, *J *= 2.1 Hz, 1H), 8.48 (d, *J *= 2.1 Hz, 1H), 8.64 (br s, 1H); δ_C_ (CDCl_3_) 109.0, 112.3, 116.9, 126.3, 127.3, 128.7, 129.8, 131.3, 133.8, 136.1, 137.3, 149.5, 177.0; *m/z* 380 (100%, C_15_H_10_NO^79^Br^81^Br^+^); HRMS (ES): MH^+^, found 377.9128. C_15_H_10_NO^79^Br_2_^+^ requires 377.9129.

*6,8-Dibromo-2-(4-fluorophenyl)-4-methoxyquinoline* (**4b**) *and 6,8-dibromo-2-(4-fluorophenyl)quinolin-4(1H)-one* (**5b**). A stirred mixture of **3b** (0.50 g, 1.26 mmol) and iodine (0.79 g, 3.14 mmol) in methanol (20 mL) afforded **4b** and **5b** in sequence.

*6,8-Dibromo-2-(4-fluorophenyl)-4-methoxyquinoline* (**4b**). White solid (0.32 g, 63%), R_f_ 0.80.

6*,8-Dibromo-2-(4-fluorophenyl)quinolin-4(1H)-one* (**5b**). White solid (0.05 g, 10%), m.p. 225–227 °C, *R*_f_ 0.50; ν_max_ (ATR) 719, 831, 1221, 1490, 1543, 1579, 1614, 3388 cm^−1^; δ_H_ (CDCl_3_) 6.54 (s, 1H), 7.28 (t, *J *= 8.4 Hz, 2H), 7.69 (t, *J *= 8.4 Hz, 2H), 7.97 (d, *J *= 2.1 Hz, 1H), 8.47 (d, *J *= 2.1 Hz, 1H), 8.56 (br s, 1H); δ_C_ (CDCl_3_) 109.0, 112.3, 117.0, 117.1 (d, ^2^*J*_CF_ = 22.2 Hz), 127.2, 128.5 (d, ^3^*J*_CF_ = 8.6 Hz), 128.7, 129.9 (d, ^4^*J*_CF_ = 3.4 Hz), 136.0, 137.4, 148.6, 164.5 (d, ^1^*J*_CF_ = 251.6 Hz), 176.9; *m/z* 396 (100, MH^+^); HRMS (ES): MH^+^, found 395.9035. C_15_H_9_NFO^79^Br_2_^+^ requires 395.9031.

*6,8-Dibromo-2-(4-chlorophenyl)-4-methoxyquinoline* (**4c**) *and 6,8-dibromo-2-(4-chlorophenyl)quinolin-4(1H)-one* (**5c**). A stirred mixture of **3c** (0.50 g, 1.20 mmol) and iodine (0.77 g, 3.02 mmol) in methanol (20 mL) afforded **4c** and **5c** in sequence.

*6,8-Dibromo-2-(4-chlorophenyl)-4-methoxy-quinoline* (**4c**). White solid (0.32 g, 64%), R_f_ 0.85.

*6,8-Dibromo-2-(4-chlorophenyl)quinolin-4(1H)-one* (**5c**). White solid (0.05 g, 9%), m.p. 237–239 °C, *R*_f_ 0.29; ν_max_ (ATR) 825, 1095, 1383, 1491, 1543, 1618, 3385 cm^−1^; δ_H_ (CDCl_3_) 6.55 (s, 1H), 7.55 (d, *J *= 8.4 Hz, 2H), 7.63 (d, *J *= 8.4 Hz, 2H), 7.96 (d, *J *= 2.1 Hz, 1H), 8.45 (d, *J *= 2.1 Hz, 1H), 8.56 (br s, 1H); δ_C_ (CDCl_3_) 109.2, 112.3, 117.1, 127.3, 127.7, 128.8 (2 × C), 130.1, 132.3, 136.0, 137.5, 148.4, 176.9; *m/z* 414 (100%, C_15_H_9_NO^37^Cl^79^Br_2_^+^); HRMS (ES): MH^+^, found 411.8737. C_15_H_9_NO^35^Cl ^79^Br_2_^+^ requires 411.8739.

*6,8-Dibromo-4-methoxy-2-(4-methoxyphenyl)quinoline* (**4d**) *and 6,8-dibromo-2-(4-methoxyphenyl)-quinolin-4(1H)-one* (**5d**). A stirred mixture of **3d** (0.50 g, 1.22 mmol) and iodine (0.77 g, 3.04 mmol) in methanol (20 mL) afforded **4d** and **5d** in sequence.

*6,8-Dibromo-4-methoxy-2-(4-methoxyphenyl)quinoline* (**4d**). White solid (0.33 g, 65%), R_f_ 0.74.

*6,8-Dibromo-2-(4-methoxyphenyl)quinolin-4(1H)-one* (**5d**). White solid (0.04 g, 8%), m.p. 200–201 °C, *R*_f_ 0.26; ν_max_ (ATR) 719, 824, 1030, 1179, 1250, 1490, 1510, 1609, 3388 cm^−1^; δ_H_ (CDCl_3_) 3.88 (s, 3H), 6.54 (s, 1H), 7.06 (d, *J *= 8.7 Hz, 2H), 7.62 (d, *J *= 8.7 Hz, 2H), 7.93 (d, *J *= 2.1 Hz, 1H), 8.45 (d, *J *= 2.1 Hz, 1H), 8.59 (br s, 1H); δ_C_ (CDCl_3_) 55.6, 108.1, 112.2, 115.1, 116.7, 125.8, 127.2, 127.7, 128.7, 136.0, 137.1, 149.3, 162.1, 176.9; *m/z* 410 (100%, C_16_H_12_NO_2_^79^Br^81^Br^+^); HRMS (ES): MH^+^, found 407.9236. C_16_H_12_NO_2_^79^Br_2_^+^ requires 407.9235.

### 3.6. Typical Procedure for the Suzuki-Miyaura Cross Coupling of *3* with Arylvinylboronic Acids

*6,8-Bis[2-(4-fluorophenyl)ethenyl]-4-methoxy-2-phenylquinoline *(**6a**). A mixture of **4a** (0.35 g, 0.892 mmol), 4-fluorophenylvinylboronic acid (0.37 g, 2.23 mmol), PdCl_2_(PPh_3_)_2_ (0.031 g, 0.045 mmol), PCy_3_ (0.025 g, 0.089 mmol) and K_2_CO_3_ (0.246 g, 1.178 mmol) in dioxane/water (20 mL) in a two-necked flask equipped with a stirrer bar, rubber septum and a condenser was flushed for 20 minutes with argon gas. A balloon filled with argon gas was then connected to the top of the condenser and the mixture was heated with stirring at 80–90 °C under argon atmosphere for 3 h. The mixture was allowed to cool to room temperature and then poured into an ice-cold water. The resulting precipitate was filtered and taken-up into chloroform. The organic solution was washed with brine, dried over anhydrous MgSO_4_, filtered and then evaporated under reduced pressure to afford **6a** as a yellow solid (0.25 g, 60%), m.p. 201–203 °C (ethanol); ν_max_ (ATR) 667, 769, 814, 831, 852, 961, 876, 1044, 1207, 1380, 1483, 1575, 1576, 1590 cm^−1^; δ_H_ (CDCl_3_) 4.13 (s, 3H), 7.08 (t, *J *= 8.7 Hz, 2H), 7.10 (t, *J *= 8.7 Hz, 2H), 7.18 (d, *J *= 16.5 Hz, 1H), 7.23 (s, 1H), 7.27 (d, *J *= 16.5 Hz, 1H), 7.42 (d, *J *= 16.5 Hz, 1H), 7.47–7.58 (m, 5H), 7.65 (t, *J *= 8.7 Hz, 2H), 8.14 (d, *J *= 1.8 Hz, 1H), 8.17 (d, *J *= 1.8 Hz, 1H), 8.23 (dd, *J *= 1.5 and 8.4 Hz, 2H), 8.48 (d, *J *= 16.5 Hz, 1H); δ_C_ (CDCl_3_) 55.7, 98.9, 115.6 (d, ^2^*J*_CF_ = 21.4 Hz), 115.7 (d, ^2^*J*_CF_ = 21.4 Hz), 119.2, 120.9, 123.5, 125.1, 127.4,128.0, 128.1 (d, ^3^*J*_CF_ = 8.0 Hz), 128.2, 128.4 (d, ^3^*J*_CF_ = 8.3 Hz), 128.8, 129.0, 129.4, 133.4 (d, ^4^*J*_CF_ = 3.5 Hz), 133.8, 134.2 (d, ^4^*J*_CF_ = 3.5 Hz), 135.6, 140.1, 146.4, 156.9, 162.4 (d, ^1^*J*_CF_ = 245.6 Hz), 162.4 (d, ^1^*J*_CF_ = 246.2 Hz), 162.8; *m/z* 476 (100, MH^+^); HRMS (ES): MH^+^, found 476.1823. C_32_H_24_NOF_2_^+^ requires 476.1826.

*6,8-Bis[2-(4-fluorophenyl)ethenyl]-2-(4-fluorophenyl)-4-methoxyquinoline *(**6b**). Yellow solid (0.28 g, 68%), m.p. 182–183 °C (ethanol); ν_max_ (ATR) 717, 825, 1001, 1218, 1360, 1438, 1481, 1542, 1582 cm^−1^; δ_H_ (CDCl_3_) 4.15 (s, 3H), 7.08 (t, *J *= 8.7 Hz, 2H), 7.11 (t, *J *= 8.7 Hz, 2H), 7.19 (s, 1H), 7.20 (d, *J *= 16.5 Hz, 1H), 7.22 (t, *J *= 8.7 Hz, 2H), 7.29 (d, *J *= 16.5 Hz, 1H), 7.42 (d, *J *= 16.5 Hz, 1H), 7.56 (t, *J *= 8.7 Hz, 2H), 7.65 (t, *J *= 8.7 Hz, 2H), 8.17 (d, *J *= 1.8 Hz, 1H), 8.20 (d, *J *= 8.7 Hz, 2H), 8.23 (s, 1H), 8.43 (d, *J *= 16.5 Hz, 1H); δ_C_ (CDCl_3_) 55.7, 97.6, 115.6 (d, ^2^*J*_CF_ = 21.3 Hz), 115.7 (d, ^2^*J*_CF_ = 21.6 Hz), 115.8 (d, ^2^*J*_CF_ = 21.6 Hz), 119.3, 120.8, 123.7, 125.0, 128.1 (d, ^3^*J*_CF_ = 8.0 Hz), 128.2 (2 × C), 128.4 (d, ^3^*J*_CF_ = 8.0 Hz), 129.2, 129.3 (d, ^3^*J*_CF_ = 8.6 Hz), 133.4 (d, ^4^*J*_CF_ = 3.5 Hz), 133.9, 134.1 (d, ^4^*J*_CF_ = 3.2 Hz), 135.6, 136.3 (d, ^4^*J*_CF_ = 3.0 Hz), 146.4, 155.9, 162.3 (d, ^1^*J*_CF_ = 245.3 Hz), 162.4 (d, ^1^*J*_CF_ = 245.9 Hz), 163.0, 163.8 (d, ^1^*J*_CF_ = 247.3 Hz); *m/z* 412 (100, MH^+^); HRMS (ES): MH^+^, found 411.9178. C_32_H_23_NOF_3_^+^ requires 411.9178.

*2-(4-Chlorophenyl)-6,8-bis[2-(4-fluorophenyl)ethenyl]-4-methoxyquinoline *(**6c**). Yellow solid (0.36 g, 88%), m.p. 228–229 °C (ethanol); ν_max_ (ATR) 816, 849, 1010, 1221, 1251, 1557, 1575 cm^−1^; δ_H_ (CDCl_3_) 4.13 (s, 3H), 7.08 (t, *J *= 8.7 Hz, 2H), 7.11 (t, *J *= 8.7 Hz, 2H), 7.17 (s, 1H), 7.18 (d, *J *= 16.5 Hz, 1H), 7.27 (d, *J *= 16.5 Hz, 1H), 7.41 (d, *J *= 16.5 Hz, 1H), 7.51(d, *J *= 9.0 Hz, 2H), 7.54 (t, *J *= 8.7 Hz, 2H), 7.64 (t, *J *= 8.7 Hz, 2H), 8.14 (s, 1H), 8.15 (d, *J *= 8.7 Hz, 2H), 8.18 (s, 1H), 8.43 (d, *J *= 16.5 Hz, 1H); δ_C_ (CDCl_3_) 55.7, 97.5, 115.6 (d, ^2^*J*_CF_ = 21.4 Hz), 115.7 (d, ^2^*J*_CF_ = 21.6 Hz), 119.2, 130.0, 123.7, 124.9, 128.1 (d, ^3^*J*_CF_ = 8.0 Hz), 128.2 (d, ^2^*J*_CF_ = 8.0 Hz), 128.4, 128.7, 128.8, 128.9, 129.2, 133.4 (d, ^4^*J*_CF_ = 3.1 Hz), 134.0, 134.1 (d, ^4^*J*_CF_ = 3.5 Hz), 135.5, 135.6, 138.5, 146.3, 155.6, 162.3 (d, ^1^*J*_CF_ = 245.3 Hz), 162.5 (d, ^1^*J*_CF_ = 246.2 Hz), 163.0; *m/z* 510 (100, MH^+^); HRMS (ES): MH^+^, found 510.1428. C_32_H_23_NO^35^ClF_2_^+^ requires 510.1436.

*6,8-Bis[2-(4-fluorophenyl)ethenyl]-4-methoxy-2-(4-methoxyphenyl)quinoline *(**6d**). Yellow solid (0.36 g, 86%), m.p. 224-225 °C (ethanol); ν_max_ (ATR) 777, 792, 852, 939, 961, 1209, 1228, 1292, 1483, 1507, 1558, 1601 cm^−1^; δ_H_ (CDCl_3_) 3.90 (s, 3H), 4.12 (s, 3H), 7.06 (d, *J *= 9.0 Hz, 2H), 7.08 (t, *J *= 8.7 Hz, 2H), 7.10 (t, *J *= 8.7 Hz, 2H), 7.18 (s, 1H), 7.17 (d, *J *= 16.5 Hz, 1H), 7.25 (d, *J *= 16.5 Hz, 1H), 7.41 (d, *J *= 16.5 Hz, 1H), 7.54 (t, *J *= 8.7 Hz, 2H), 7.65 (t, *J *= 8.7 Hz, 2H), 8.13 (s, 1H), 8.15 (s, 1H), 8.19 (d, *J *= 9.0 Hz, 2H), 8.46 (d, *J *= 16.5 Hz, 1H); δ_C_ (CDCl_3_) 55.4, 55.7, 97.3, 114.5, 115.6 (d, ^2^*J*_CF_ = 21.7 Hz), 115.7 (d, ^2^*J*_CF_ = 21.7 Hz), 119.3, 120.7, 123.6, 125.3, 127.9, 128.0 (d, ^3^*J*_CF_ = 8.0 Hz), 128.3 (d, ^3^*J*_CF_ = 8.0 Hz), 128.4, 128.7, 128.9, 132.7, 133.4, 133.5 (d, ^4^*J*_CF_ = 3.4 Hz), 134.3 (d, ^4^*J*_CF_ = 3.4 Hz), 135.4, 146.5, 156.6, 160.8, 162.3 (d, ^1^*J*_CF_ = 245.3 Hz), 162.4 (d, ^1^*J*_CF_ = 245.8 Hz), 162.7; *m/z* 506 (100, MH^+^); HRMS (ES): MH^+^, found 506.1932. C_33_H_26_NO_2_F_2_^+^ requires 506.1932.

*6,8-Bis[2-(4-chlorophenyl)ethenyl]-4-methoxy-2-phenylquinoline *(**6e**). Yellow solid (0.31 g, 69%), m.p. 187–188 °C (ethanol); ν_max_ (ATR) 698, 777, 810, 903, 1011, 1272, 1288, 1360, 1489, 1579 cm^−1^; δ_H_ (CDCl_3_) 4.13 (s, 3H), 7.22 (s, 1H), 7.23 (s, 2H), 7.35 (d, *J *= 8.7 Hz, 2H), 7.37 (d, *J *= 8.7 Hz, 2H), 7.40 (d, *J *= 16.5 Hz, 1H), 7.49 (d, *J *= 8.7 Hz, 2H), 7.54 (d, *J *= 8.7 Hz, 2H), 7.55 (d, *J *= 16.5 Hz, 1H), 7.60 (d, *J *= 8.7 Hz, 2H), 8.15 (d, *J *= 1.8 Hz, 1H), 8.16 (d, *J *= 1.8 Hz, 1H), 8.20 (dd, *J *= 1.8 and 8.4 Hz, 2H), 8.53 (d, *J *= 16.5 Hz, 1H); δ_C_ (CDCl_3_) 55.7, 97.9, 119.67, 120.9, 123.7, 126.0, 127.5, 127.7, 128.0 (2 × C), 128.7, 128.8, 128.9, 129.0, 129.1, 129.5, 133.1, 133.3, 133.6, 135.5, 135.7, 136.5, 140.1, 146.5, 157.1, 162.9; *m/z* 508 (100, MH^+^); HRMS (ES): MH^+^, found 508.1247. C_32_H_24_NO^35^Cl_2_^+^ requires 508.1235.

*6,8-Bis[2-(4-chlorophenyl)ethenyl]-2-(4-fluorophenyl)-4-methoxyquinoline *(**6f**). Yellow solid (0.36 g, 81%), m.p. 213–214 °C (ethanol); ν_max_ (ATR) 813, 826, 839, 860, 1217, 1308, 1511, 1589 cm^−1^; δ_H_(CDCl_3_) 4.12 (s, 3H), 7.15 (s, 1H), 7.22 (d, *J *= 8.4 Hz, 2H), 7.23 (t, *J *= 8.7 Hz, 2H), 7.35 (d, *J *= 16.5 Hz, 2 × H), 7.36 (t, *J *= 8.7 Hz, 2H), 7.37 (d, *J *= 16.5 Hz, 1H), 7.49 (d, *J *= 8.7 Hz, 2H), 7.59 (d, *J *= 8.4 Hz, 2H), 8.13 (d, *J *= 1.8 Hz, 1H), 8.15 (d, *J *= 1.8 Hz, 1H), 8.19 (t, *J *= 8.7 Hz, 2H), 8.48 (d, *J *= 16.5 Hz, 1H); δ_C_ (CDCl_3_) 55.7, 97.6, 115.6 (d, ^2^*J*_CF_ = 21.4 Hz), 119.6, 120.8, 123.8, 125.8, 127.7, 128.0, 128.1, 128.8, 128.9, 129.0, 129.2, (d, ^3^*J*_CF_ = 8.3 Hz), 133.2, 133.4, 133.7, 135.4, 135.7, 136.2 (d, ^4^*J*_CF_ = 3.1 Hz), 136.4, 146.4, 155.9, 162.9, 163.8 (d, ^1^*J*_CF_ = 247.6 Hz); *m/z* 526 (100, MH^+^); HRMS (ES): MH^+^, found 526.1143. C_32_H_23_NO^35^Cl_2_F^+^ requires 526.1141.

*2-(4-Chlorophenyl)-6,8-bis[2-(4-chlorophenyl)ethenyl]-4-methoxyquinoline* (**6g**). Yellow solid (0.28 g, 64%), m.p. 217–219 °C (ethanol); ν_max_ (ATR) 822, 846, 969, 1012, 1151, 1161, 1211, 1361, 1491, 1591 cm^−1^; δ_H_ (DMSO-*d*_6_) 4.18 (s, 3H), 7.47 (d, *J *= 8.7 Hz, 2H), 7.52 (s, 4H), 7.60 (d, *J *= 8.4 Hz, 2H), 7.65 (s, 1H), 7.67 (d, *J *= 16.5 Hz, 1H), 7.71 (d, *J *= 8.7 Hz, 2H), 7.73 (d, *J *= 8.7 Hz, 2H), 8.14 (d, *J *= 1.5 Hz, 1H), 8.40 (d, *J *= 8.7 Hz, 2H), 8.44 (d, *J *= 16.5 Hz, 1H), 8.48 (d, *J *= 1.5 Hz, 1H); δ_C_ (DMSO-*d*_6_) 56.8, 99.0, 120.2, 120.9, 124.1, 125.8, 128.5, 128.6, 128.7, 128.8, 129.2 (2 × C), 129.4, 129.5, 129.6 (2xC), 132.6, 134.3, 134.9, 135.2, 136.5, 137.0, 138.5, 145.9, 155.5, 163.1; *m/z* 542 (100, MH^+^); HRMS (ES): MH^+^, found 542.0842. C_32_H_23_NO^35^Cl_3_^+^ requires 542.0845.

*6,8-Bis[2-(4-chlorophenyl)ethenyl]-4-methoxy-2-(4-methoxyphenyl)quinoline* (**6h**). Yellow solid (0.26 g, 60%), m.p. 244–246 °C (ethanol); ν_max_ (ATR) 785.7, 805, 957, 975, 1170, 1215, 1292, 1363, 1489, 1588 cm^−1^; δ_H_ (CDCl_3_) 3.90 (s, 3H), 4.12 (s, 3H), 7.06 (d, *J *= 8.7 Hz, 2H), 7.10 (d, *J *= 16.5 Hz, 1H), 7.17 (s, 1H), 7.22 (s, 2H), 7.36 (d, *J *= 16.5 Hz, 1H), 7.37 (d, *J *= 8.4 Hz, 2H), 7.39 (d, *J *= 16.5 Hz, 1H), 7.49 (d, *J *= 8.7 Hz, 2H), 7.60 (d, *J *= 8.4 Hz, 2H), 8.13 (d, *J *= 1.8 Hz, 1H), 8.14 (d, *J *= 1.8 Hz, 1H), 8.18 (d, *J *= 8.7 Hz, 2H), 8.51 (d, *J *= 16.5 Hz, 1H); δ_C_ (CDCl_3_) 55.4, 55.7, 97.4, 114.2, 119.7, 120.7, 123.7, 126.1, 127.7, 127.8, 127.9, 128.0, 128.7, 128.8, 128.9, 129.1, 132.7, 133.1, 133.2, 133.3, 135.3, 135.8, 136.6, 146.6, 156.7, 160.9, 162.8; *m/z* 538 (100, MH^+^); HRMS (ES): MH^+^, found 538.1348. C_33_H_26_NO_2_^35^Cl_2_^+^ requires 538.1341.

*4-Methoxy-6,8-bis[2-(4-methoxyphenyl)ethenyl]-2-phenylquinoline *(**6i**). Green solid (0.28 g, 63%), m.p. 195–196 °C (ethanol); ν_max_ (ATR) 696, 775, 953, 1035, 1286, 1303, 1508, 1575, 1589, 1604 cm^−1^; δ_H_ (CDCl_3_) 3.85 (s, 3H), 3.86 (s, 3H), 4.14 (s, 3H), 6.94 (t, *J *= 8.7 Hz, 2H), 6.95 (t, *J *= 8.7 Hz, 2H), 7.16 (d, *J *= 16.5 Hz, 1H), 7.24 (d, *J *= 16.5 Hz, 1H), 7.25 (s, 1H), 7.28 (d, *J *= 16.5 Hz, 1H), 7.44 (d, *J *= 16.5 Hz, 1H), 7.53 (d, *J *= 8.7 Hz, 2 × 2H), 7.64 (d, *J *= 8.7 Hz, 2H), 8.12 (d, *J *= 1.5 Hz, 1H), 8.20 (d, *J *= 1.5 Hz, 1H), 8.24 (dd, *J *= 1.8 and 8.4 Hz, 2H), 8.46 (d, *J *= 16.5 Hz, 1H); δ_C_ (CDCl_3_) 55.3 (2 × C), 55.6, 97.7, 114.1, 114.2, 118.4, 120.9, 123.2, 123.3, 126.5, 127.4, 127.8, 128.1, 128.7, 128.8, 129.2, 129.6, 130.1, 131.0, 134.3, 135.9, 140.3, 146.2, 156.4, 159.2, 157.4, 162.8; *m/z* 500 (100, MH^+^); HRMS (ES): MH^+^, found 500.2237. C_34_H_30_NO_3_^+^ requires 500.2235.

*2-(4-Fluorophenyl)-4-methoxy-6,8-bis[2-(4-methoxyphenyl)ethenyl]quinoline *(**6j**). Green solid (0.27 g, 60%), m.p. 180–181 °C (ethanol); ν_max_ (ATR) 788, 957, 1173, 1305, 1486, 1509, 1584, 1602 cm^−1^; δ_H_ (CDCl_3_) 3.84 (s, 3H), 3.86 (s, 3H), 4.07 (s, 3H), 6.92 (d, *J *= 8.7 Hz, 2H), 6.95 (d, *J *= 8.7 Hz, 2H), 7.08 (s, 1H), 7.11 (d, *J *= 16.5 Hz, 1H), 7.20 (t, *J *= 8.7 Hz, 2H), 7.23 (d, *J *= 16.5 Hz, 1H), 7.28 (d, *J *= 16.5 Hz, 1H), 7.51 (d, *J *= 8.7 Hz, 2H), 7.62 (d, *J *= 8.7 Hz, 2H), 8.03 (d, *J *= 1.5 Hz, 1H), 8.13 (d, *J *= 1.5 Hz, 1H), 8.19 (t, *J *= 8.7 Hz, 2H), 8.38 (d, *J *= 16.5 Hz, 1H); δ_C_ (CDCl_3_) 55.3 (2 × C), 55.6, 57.2, 114.1, 114.2, 115.5 (d, ^2^*J*_CF_ = 21.3 Hz), 118.3, 120.8, 123.1, 123.3, 126.4, 127.8, 128.1, 128.8, 129.2, (d, ^3^*J*_CF_ = 8.3 Hz), 129.6, 130.1, 130.9, 134.3, 135.8, 136.3 (d, ^4^*J*_CF_ = 3.0 Hz), 146.1, 155.2, 159.3, 159.4, 162.8, 163.7 (d, ^1^*J*_CF_ = 247.1 Hz); *m/z* 518 (100, MH^+^); HRMS (ES): MH^+^, found 518.2136. C_34_H_29_NO_3_F^+^ requires 518.2131.

*2-(4-Chlorophenyl)-4-methoxy-6,8-bis[2-(4-methoxyphenyl)ethenyl]quinoline *(**6k**). Green solid (0.26 g, 60%), m.p. 201–203 °C (ethanol); ν_max_ (ATR) 844, 899, 1011, 1083, 1129, 1304, 1461, 1484, 1574, 1604 cm^−1^; δ_H_ (CDCl_3_) 3.85 (s, 3H), 3.86 (s, 3H), 4.12 (s, 3H), 6.95 (d, *J *= 8.7 Hz, 2H), 6.96 (d, *J *= 8.7 Hz, 2H), 7.14 (d, *J *= 16.5 Hz, 1H), 7.16 (s, 1H), 7.27 (d, *J *= 16.5 Hz, 1H), 7.43 (d, *J *= 16.5 Hz, 1H), 7.51 (d, *J *= 8.7 Hz, 2H), 7.52 (d, *J *= 8.7 Hz, 2H), 7.63 (d, *J *= 8.7 Hz, 2H), 8.09 (d, *J *= 1.5 Hz, 1H), 8.15 (d, *J *= 1.5 Hz, 1H), 8.16 (d, *J *= 8.7 Hz, 2H), 8.39 (d, *J *= 16.5 Hz, 1H); δ_C_ (CDCl_3_) 55.3 (2 × C), 55.7, 97.3, 114.1, 114.2, 118.4, 121.0, 123.1, 123.4, 126.4, 127.8, 128.1, 128.7, 128.9, 129.0, 129.8, 130.1, 130.9, 134.6, 135.3, 136.0, 138.7, 146.2, 155.1, 159.3, 159.4, 162.9; *m/z* 534 (100, MH^+^); HRMS (ES): MH^+^, found 534.1829534.1829. C_34_H_29_NO_3_^35^Cl^+^ requires 534.1836.

*4-Methoxy-2-(4-methoxyphenyl)-6,8-bis[2-(4-methoxyphenyl)ethenyl]quinoline* (**6l**). Green solid (0.28 g, 65%), m.p. 181–182 °C (ethanol); ν_max_ (ATR) 807.3, 1027, 1102, 1244, 1303, 1361, 1508, 1605 cm^−1^; δ_H_ (CDCl_3_) 3.85 (s, 3H), 3.87 (s, 3H), 3.91 (s, 3H), 4.11 (s, 3H), 6.93 (d, *J *= 8.7 Hz, 2H), 6.96 (d, *J *= 8.7 Hz, 2H), 7.07 (d, *J *= 8.7 Hz, 2H), 7.14 (d, *J *= 16.2 Hz, 1H), 7.15 (s, 1H), 7.26 (d, *J *= 16.2 Hz, 1H), 7.43 (d, *J *= 16.5 Hz, 1H), 7.53 (d, *J *= 8.7 Hz, 2H), 7.65 (d, *J *= 8.7 Hz, 2H), 8.07 (d, *J *= 1.5 Hz, 1H), 8.16 (d, *J *= 1.5 Hz, 1H), 8.21 (d, *J *= 8.7 Hz, 2H), 8.44 (d, *J *= 16.5 Hz, 1H); δ_C_ (CDCl_3_) 55.3 (2 × C), 55.4, 55.6, 97.1, 114.0, 114.1, 114.2, 118.4, 120.7, 123.3, 123.5, 126.6, 127.8, 128.1, 128.6, 128.7, 129.5, 130.2, 131.1, 132.9, 134.0, 135.8, 146.3, 156.0, 159.3, 159.4, 160.8, 162.7; *m/z* 530 (100, MH^+^); HRMS (ES): MH^+^, found 530.1842. C_35_H_32_NO_4_^+^ requires 530. 1842.

### 3.7. Crystal Structure Solution and Refinement

X-ray quality crystals of the title compound **3b** were obtained by slow crystallization from ethanol solution. Intensity data were collected on a Bruker APEX II CCD area detector diffractometer with graphite monochromated Mo *K*_α_ radiation (50 kV, 30 mA) using the Bruker APEX 2 [39] data collection software. The collection method involved ω-scans of width 0.5° and 512 × 512 bit data frames. Data reduction was carried out using the program Bruker SAINT*+* [40]. The crystal structure was solved by direct methods using Bruker SHELXTL [41]. Non-hydrogen atoms were first refined isotropically followed by anisotropic refinement by full matrix least-squares calculations based on *F*^2^ using *SHELXTL*. Hydrogen atoms were first located in the difference map then positioned geometrically and allowed to ride on their respective parent atoms. Diagrams and publication material were generated using SHELXTL, PLATON [42] and ORTEP-3 [43]. Crystallographic parameters are summarized in Table 1. 

**Table 1:** Crystal data and structure refinement for compound **3b**.

Empirical formula	C_15_H_10_Br_2_FNO
Formula weight	399.06
Temperature	173(2) K
Wavelength	0.71073 Å
Crystal system	Monoclinic
Space group	P2(1)/n
Unit cell dimensions	a = 13.0752(6) Å α = 90°b = 8.0086(3) Å β = 111.8230(10)°c = 14.3026(6) Å γ = 90°
Volume	1390.35(10) Å^3^
Z	4
Density (calculated)	1.906 Mg/m^3^
Absorption coefficient	5.835 mm^−1^
F(000)	776
Crystal size	0.41 × 0.40 × 0.20 mm^3^
Theta range for data collection	1.80 to 27.99°.
Index ranges	−17 ≤ h ≤ 17, −10 ≤ k ≤ 10, −18 ≤ l ≤ 16
Reflections collected	17097
Independent reflections	3358 [R(int) = 0.0768]
Completeness to theta = 27.00°	99.9%
Absorption correction	Integration
Max. and min. transmission	0.3882 and 0.1983
Refinement method	Full-matrix least-squares on F^2^
Data / restraints / parameters	3358 / 0 / 185
Goodness-of-fit on F^2^	0.922
Final R indices [I>2sigma(I)]	R1 = 0.0277, wR2 = 0.0561
R indices (all data)	R1 = 0.0391, wR2 = 0.0584
Largest diff. peak and hole	0.470 and −0.709 e.Å^−3^

## 4. Conclusions

The generality, brevity and operational simplicity of iodine/methanol–mediated oxidative aromatization reaction of the 2-aminochalcones or their 2,3-dihydroquinolin-4(1*H*)-one isomers and the accompanying high yields make this methodology a suitable alternative to metal–catalyzed aromatization and subsequent methylation or oxidative aromatization–dechloromethoxylation of related derivatives. Elaboration of the 6,8-dibromoquinoline scaffold via Suzuki-Miyaura cross-coupling with arylvinylboronic acids, on the other hand, afforded polysubstituted quinoline derivatives that would not be easily accessible via the known classical methods such as the Skraup, Friedlander and Doebner-von Miller reactions. The absorption and fluorescent properties of these compounds showed a strong correlation with the substituent groups on the styryl and the 2-phenyl groups with the halo substituents shifting both the absorption and emission maxima to shorter wavelengths. Many styrylquinolines prove to be dyes with enhanced photo- and electroluminescent properties and they find application in medicine and pharmacology [44]. The preliminary photophysical properties of compounds **6** serve as a prelude to compounds with potential photonic or electronic properties.
